# Utility of *Bartonella henselae* Recombinant Proteins ATP-β and GroEL for Serodiagnosis of Feline Bartonellosis

**DOI:** 10.3390/ani16213527

**Published:** 2026-11-07

**Authors:** Pradeep Neupane, Jessika Bryant, Emily Kingston, Ricardo G. Maggi, Marcos Rogério André, Edward B. Breitschwerdt

**Affiliations:** 1 Department of Clinical Sciences and the Intracellular Pathogens Research Laboratory, Comparative Medicine Institute, College of Veterinary Medicine, North Carolina State University, 1060 William Moore Dr., Raleigh, NC 27607, USA; pneupan@ncsu.edu (P.N.); jgrindstaff1996@gmail.com (J.B.); emkingst@ncsu.edu (E.K.); rgmaggi@ncsu.edu (R.G.M.); 2 Vector-Borne Bioagents Laboratory (VBBL), Department of Pathology, Reproduction and One Health, Faculdade de Ciências Agrárias e Veterinárias, Universidade Estadual Paulista (FCAV/UNESP), Jaboticabal 14884-900, SP, Brazil; mr.andre@unesp.br

**Keywords:** serology, diagnosis, ELISA, antigen, *Bartonella henselae*, IFA

## Abstract

*Bartonella* species are small, fastidious, Gram-negative intracellular bacteria that are capable of infecting a wide range of mammals, including cats, dogs and humans. Diagnoses of canine, feline, and human bartonellosis by culture, molecular, and serological assays remains challenging. Accurate serodiagnosis of feline bartonellosis is of clinical, epidemiological and zoonotic importance. The utility of *Bartonella henselae* (*Bh*) ATP-β, ATP synthase beta subunit, and GroEL, a chaperonin protein, has not previously been reported for diagnosis of feline bartonellosis. The objective of this study was to assess the diagnostic utility of *Bh* recombinant proteins ATP-β and GroEL using sera samples from Group I: four cats experimentally inoculated with *Bh* or saline (*n* = 24); Group II: 34 cats naturally infected with *Bh* (*n* = 34)*;* Group III: 33 *Bartonella* spp. qPCR-negative and IFA-negative control cats (*n* = 33), and Group IV: 16 *Mycoplasma haemofelis* qPCR-positive cats (*n* = 16). Based on the *Bh* rATP-β, rGroEL, and rATP-β plus rGroEL ELISA results, rGroEL yielded the highest area under the receiver operating characteristic curve (AUC) of 0.84 (95% CI 0.74–0.94), followed by rATP-β plus rGroEL with an AUC of 0.77 (95% CI 0.65–0.89). The sensitivity and specificity of rGroEL were 50% (95% CI 34–66%) and 91% (95% CI 76–97%), respectively, at an OD cutoff value of 0.1, whereas the sensitivity and specificity of rATP-β plus rGroEL ELISA (combination of rATP-β and rGroEL) were 71% (95% CI 54–83%) and 85% (95% CI 69–93%), respectively, at an OD cutoff value of 0.17. Overall agreement between *Bh* IFA and rGroEL-based ELISA was 70% (95% CI 58–80%), and overall agreement between *Bh* IFA and rATP-β plus rGroEL-based ELISA was 78% (95% CI 66–86%). The rATP-β-based ELISA had a poorer AUC (0.61 (95% CI 0.47–0.75)), indicating limited clinical utility for the serodiagnosis of *Bh* infection in cats. These findings suggest that rGroEL and rATP-β plus rGroEL may serve as reliable ELISA antigens for serodiagnosis of *Bartonella* infection in cats. Limitations of this study include the small sample size, the moderate sensitivity of ELISA, and its incomplete agreement with *Bh* IFA and PCR results. Future epitope mapping and bioinformatic screening of the antigenic regions of rATP-β and rGroEL may facilitate the development of a faster, more practical, and more cost-effective alternative to the labor-intensive reference IFA for the serodiagnosis of feline bartonellosis.

## Simple Summary

Cats infected with *Bartonella* species, including *B. henselae,* may develop clinical manifestations, ranging from mild, self-limiting to potentially life-threatening conditions, such as endocarditis, myocarditis, uveitis, and lymphadenopathy. Because cats can transmit zoonotic *Bartonella* species to humans, these infections are also an important public health concern. Current diagnostic methods for cats, including culture, PCR, and serological assays, have limitations that affect diagnostic accuracy and reliability. In this study, we evaluated two *B. henselae* proteins, rATP-β and rGroEL, in serological assays for detecting antibodies against *B. henselae* in cats. Using serum samples from infected and uninfected cats, we found that the rGroEL-based assay showed high specificity but detected only half of the infected cats, whereas the rATP-β-based assay showed limited diagnostic utility for detecting *B. henselae* infection or exposure. These findings suggest that rGroEL is a promising candidate for a test used alongside other diagnostic methods. However, further optimization and validation in larger groups of cats are needed to improve their accuracy and reliability in clinical settings.

## 1. Introduction

*Bartonella* species are small, fastidious, Gram-negative intracellular bacteria that infect humans and mammals, including cats and dogs [1,2,3,4]. Cats are the main reservoir host for three zoonotic *Bartonella* species, namely *Bartonella henselae* (*Bh*)*, B. clarridgeiae, and B. koehlerae* [1,5,6,7]. Worldwide seroepidemiologic studies have reported *Bh* seroprevalences in cats ranging from 4% to 80%, with the highest prevalences in warm, humid, and flea (*Ctenocephalides felis felis*)-infested regions [6,8,9]. Following infection, cats maintain a long-lasting relapsing bacteremia that can persist for years [6,7,10], resulting in a high prevalence of infection particularly among flea-infested feral cats. *Bartonella henselae* is the most common *Bartonella* species infecting cats, dogs and humans (cat scratch disease) [7,8,11,12]. Human bartonellosis has been associated with cardiac pathology (endocarditis, myocarditis), a spectrum of neurological presentations (headache, memory loss, neuroretinitis, psychoses, seizures and others), and rheumatological symptoms (joint pain, muscle pain, and osteomyelitis) [2,7,12,13]. *Bartonella henselae* is transmitted among cats by *C. felis felis* fleas, or by the inoculation of infected flea feces through the skin during scratching [14]. Transmission from cats to humans occurs when a *Bartonella* species is conveyed by scratches, bites, or saliva through broken skin [1,2,6]. Due to increased occupational exposures to cats, dogs and flea vectors, veterinary healthcare professionals are at high risk for human bartonellosis [1,2,15]. As emerging pathogens that induce chronic, indolent infections in immunocompromised and immunocompetent people [2,12], the transmission of zoonotic *Bartonella* species from cats to humans poses a serious public health concern. Timely diagnosis of *Bartonella*-infected cats is crucial for managing, controlling, and preventing infections in accidental human hosts.

Diagnosis of canine, feline, and human bartonellosis is obtained by culture, molecular, and serological assays, each of which has benefits and limitations [16,17,18,19]. Culturing *Bartonella* sp. from blood and other clinical specimens is time-consuming and can take up to 45 days for definitive diagnosis. Serological assays, such as indirect fluorescence assay (IFA) and enzyme-linked immunosorbent assay (ELISA) have been widely used for the diagnosis of canine, feline, and human bartonellosis [9,15,17,18,19,20]. The sensitivity of IFA, the most common serological assay, varies among laboratories, depending on the species, strain, antigen preparation, and cutoff value.

Previous studies suggest that IFA substantially underestimates the true seroprevalence of *Bartonella* spp. infection in cats [1,6,9]. A previous study investigating the prevalence of *Bartonella henselae* prevalence in Italian stray cats recommended the combined use of blood culture and serology, as only 12.2% (65/531) of cats were both bacteremic and seropositive [21]. Of these 531 cats, 50 of the 115 bacteremic cats were seronegative, whereas 139 of the 416 nonbacteremic cats were seropositive, indicating discordance between bacteremia and serological results. Another study reviewing the diagnosis of *Bartonella* infection in cats, dogs, and humans by culture, ELISA, IFA, and PCR highlighted the low sensitivity of these assays, the lack of test standardization, and discordance between, serological and molecular approaches [22]. To our knowledge, *Bh* proteins in ELISAs have not been critically investigated for their use in seroepidemiologic studies or for diagnosis of feline bartonellosis. Therefore, development and validation of a reliable ELISA is of One Health importance to access feline exposure, for disease diagnosis, and potentially human zoonotic disease risk.

Information related to immunoreactive proteins associated with *Bh* infections in cats is limited. A previous study, using large-scale analysis of *Bh* protein microarrays, identified several immunodominant antigens recognized by infected cats, including ATP-β (ATP synthase beta subunit), GroEL (chaperonin protein), VceA (a multidrug resistant protein), and LemA (a membrane protein) [23]. Other studies found that rP26 (an immunogenic protein P26) was immunogenic in cats and that a *Bh* rP26-based ELISA may represent a useful tool for diagnosis of feline exposure to infection with *Bh* [24]. Previous studies from our laboratory using sera from dogs naturally and experimentally with *Bartonella* species identified *Bh* ATP-β and GroEL as relevant immunodominant antigens [25,26]. The utility of an ATP-β- or GroEL-based ELISA has not previously been reported for feline bartonellosis. As the primary reservoir host of *Bh*, cats may develop immune responses that differ from those of dogs, which are considered incidental hosts [3,6,7,27]. The immune response and antigen recognition profile of dogs exposed to *Bartonella* spp. may not reflect those of cats. This study therefore provides a preliminary assessment of rATP-β and rGroEL as diagnostic antigens in cats. Therefore, the objective of this study was to assess the diagnostic utility of *Bh* recombinant proteins rATP-β and rGroEL for the diagnosis of feline bartonellosis.

## 2. Materials and Methods

### 2.1. Serum Samples

To assess the diagnostic utility of *Bh* recombinant proteins rATP-β and rGroEL, one hundred and seven archived sera from cats previously tested at North Carolina State University, College of Veterinary Medicine, Vector Borne Diseases Diagnostic Laboratory (NCSU-CVM-VBDDL) were selected for ELISA testing. Serum was derived from four cat groups. Samples were from Group I: 2 cats experimentally inoculated with *Bh* and 2 saline-inoculated controls, Group II: 34 qPCR-positive cats naturally infected with *Bh*, Group III: 33 *Bartonella* spp. qPCR-negative and IFA-negative cats, and Group IV: 16 *Mycoplasma haemofelis* qPCR-positive cats. Sera were submitted to the NCSU-CVM-VBDDL for diagnostic testing between 2011 and 2016, after which aliquots were stored at −80 °C after initial processing by the NCSU-CVM-VBDDL. The sample size was determined by the availability of archived samples and their *Bartonella* test results at the time of the study. Cats were considered IFA-seropositive with a titer of 1: 64 or greater, and negative with titers of ≺16, 16, or 32.

Group I (cats experimentally infected with *Bh* or saline-inoculated)

Group I consisted of twenty-four sera from four adult female cats from a previous experimental infection study [28]. There were six samples per cat; 12 samples from two cats experimentally infected with *Bh* and 12 samples from two saline-inoculated control cats. From each cat, one blood specimen was collected prior to inoculation and the remainder weekly post-inoculation (*n* = 5).Sera from the four cats experimentally inoculated with *Bh* or saline was obtained in conjunction with a study approved by High Quality Research (HQR), Fort Collins, CO, USA, under an approved protocol (number #170.059), as previously described [25].

All four cats were PCR-negative for *Bh* bacteremia prior to experimental intradermal challenge with *Bh* CSU-1 strain or saline, as described previously [28]. In all four cats prior to inoculation, *Bartonella* was not detected by BAPGM enrichment followed by qPCR/ddPCR, direct blood qPCR/ddPCR, or blood agar plate isolation, as described previously [28]. One *Bh*-inoculated and two control cats were *Bh* IFA-positive (titer of ≥1:64) prior to inoculation, as previously described [28]. Based upon enrichment blood culture, 16S-23S internal transcribed spacer (ITS) region-based qPCR and 16S-23S ddPCR, *Bh* bacteremia was confirmed for two *Bh* CSU-inoculated cats after experimental challenge [28].

Group II (34 *Bh* qPCR-positive and IFA-positive naturally infected cats)

Group II consisted of sera from 34 *Bh* IFA and qPCR-positive naturally infected cats. All cats were IFA-seropositive for *Bh* and were *Bh* bacteremic based on amplification of the ssrA gene encoding *Bartonella* transfer-messenger RNA (tmRNA).

Group III (33 *Bartonella* sp. qPCR-negative and IFA-negative cats)

Group III consisted of 33 diagnostic sera from cats selected from the NCSU-CVM-VBDDL archives. All 33 cats were previously IFA-negative (titers, ≤1:32 screening dilution) for three *Bartonella* spp. (*Bh*, *B. vinsonii* subsp. *berkhoffii*, and *B. koehlerae*). These cats were presumably unexposed to *Bartonella* spp. These cats were not seropositive to *Anaplasma*, *Borrelia burgdorferi*, and *Ehrlichia* antigens using a commercial enzyme-linked immunosorbent assay (ELISA; SNAP 4Dx^+^; Idexx Laboratories, Westbrook, MI) and were qPCR-negative for *Bartonella, Piroplasma, Ehrlichia, Anaplasma, Rickettsia,* and hemotropic *Mycoplasma* (NCSU-CVM-VBDDL, Raleigh, NC). Vector-borne diagnostic qPCR assays targeting *Anaplasma*, *Apicomplexa*, *Babesia*, *Bartonella*, *Cytauxzoon*, *Ehrlichia*, hemotropic *Mycoplasma*, and *Rickettsia* species were performed at NCSU-CVM-VBDDL, as previously described [29,30,31,32].

Group IV (16 *Mycoplasma haemofelis* qPCR-positive cats)

Group IV consisted of sera from 16 cats naturally infected with *Mycoplasma haemofelis*. All 16 cats were *Bartonella* sp. qPCR-negative and IFA-negative and *M. haemofelis* qPCR-positive. These cats were seronegative to *Anaplasma*, *Piroplasma*, *Borrelia burgdorferi*, and *Ehrlichia* antigens using a commercial enzyme-linked immunosorbent assay (ELISA; SNAP 4Dx^+^; Idexx Laboratories, Westbrook, MI) and were qPCR-negative for *Bartonella, Piroplasma, Ehrlichia, Anaplasma,* and *Rickettsia.*

### 2.2. Cloning, Expression, and Purification of Bh Recombinant ATP-β and GroEL Proteins

Selected regions of rATP-β (WP_011181298.1; 109 to 265 aa) and GroEL (WP_011181151.1; 193 to 370 aa) were cloned and expressed using a Champion^TM^ pET200 Directional TOPO^®^ Expression kit and *E. coli* BL21 (DE3) Star™ system (Invitrogen, Carlsbad, CA, USA), as previously described [25]. ATP-β and GroEL regions were selected based on bioinformatic analysis, including B-cell epitope mapping, surface accessibility, and antigenicity, as previously described [33]. Recombinant rATP-β and rGroEL were purified by column chromatography using HisPur^TM^ Cobalt spin columns (Thermo Scientific, Rockford, IL, USA) and dialyzed into phosphate-buffered saline (PBS; overnight) using Slide-A-Lyzer™ Dialysis Cassettes according to the manufacturer’s instructions (Thermo Scientific, Rockford, IL, USA), as previously described [33]. The cloning of in-frame DNA sequence encoding *Bh atp-β* and *groEL* in pET200D/TOPO expression system (Invitrogen, Carlsbad, CA, USA) was confirmed by Sanger sequencing. Purified rATP-β and rGroEL proteins were confirmed by Coomassie-stained SDS-PAGE and WB analysis as previously described [25].

### 2.3. ELISA

The diagnostic utility of purified recombinant rATP-β and rGroEL were evaluated by ELISA using sera from four cat groups: Group I (*n* = 24; experimentally infected cats with *Bh* or saline-inoculated), Group II (*n* = 34; naturally infected cats with *Bh*), Group III (*n* = 33; control group), and Group IV (*n* = 16; cats positive for *Mycoplasma haemofelis* by qPCR and negative for *Bartonella* by qPCR and IFA). Each serum sample was tested in triplicate by ELISA, as described previously [33]. In brief, each recombinant protein was immobilized in ELISA plate wells (500 ng/well) using carbonate buffer of pH 9.6. For the combined rATP-β plus rGroEL ELISA, the two proteins were mixed to a final concentration of 5 µg/mL each (10 µg/mL total), and 100 µL of the mixture (500 ng of rATP-β and 500 ng of rGroEL) was coated per well. After 4 °C overnight coating, plates were washed with 1× PBS three times and incubated with a blocking solution (1× PBS containing 3% Bio-Rad blotting grade non-fat dry milk) at room temperature (RT) for 2 h. After washing with 1× PBS containing 0.05% Tween-20 (Bio-Rad, Hercules, CA, USA) three times, plates were incubated with 100 µL of cat sera at 1:100 dilution in blocking solution at RT for 1 h, followed by five washes with 1× PBST and incubation with 100 µL of HRP-conjugated goat anti-cat IgG as a secondary antibody (1:10,000 dilution; Jackson ImmunoResearch, West Grove, PA, USA) for 1 h at RT. After washing the plates with 1× PBST for five times, 50 µL of 1-Step™ Ultra TMB-ELISA substrate solution (Invitrogen, Carlsbad, CA, USA) was added to the plate wells and incubated at RT for 15 min, followed by the addition of 2M H_2_SO_4_ to stop the reaction. The absorbance was measured at a wavelength of 450 nm using a Tecan plate reader. Sera from a cat naturally infected with *Bh* (*Bh* IFA titer = 1: 1024) and a *Bartonella* qPCR-negative and IFA-negative cat sera were used as positive and negative controls, respectively. To subtract background noise, wells containing only coating buffer (without protein) were used as blanks. After subtracting blank absorbance values, average sample absorbance values were calculated from the corresponding triplicate runs. The Youden index (*J*) was used to determine the optimal ELISA cutoff values. Based on the Youden index (*J*) and the trade-off between sensitivity and specificity, an optimal OD cutoff value was determined for each ELISA.

### 2.4. Statistical Analysis

To determine the sensitivity, specificity, and cutoff values for ELISA, receiver operating characteristic (ROC) analysis was performed with 95% CIs, as previously described [33]. Since IFA is the historical standard, and the most common serological test used for the diagnosis of *Bartonella* infection in cats, *Bh* IFA results were the comparative diagnostic assay for the calculation of ELISA sensitivity and specificity. To compare agreement between *Bh* IFA and ELISA serological results for the two groups (Group II infected and III control), the positive, negative, and overall percent agreements between ELISA and IFA were calculated as described previously [34]. Cohen’s Kappa statistic was calculated to measure the agreement between the ELISA and IFA tests, as described previously [35].

The scatterplots and ROC curve analysis for ELISA seroreactivity were generated using GraphPad Prism 11.0.1 (accessed on 15 April 2026). Differences in the ELISA IgG reactivity between the naturally infected and control groups (Group II and III) were analyzed using the Mann–Whitney U test. *p*-values of less than 0.05 were considered statistically significant.

## 3. Results

### 3.1. Reactivity of rATP-β, rGroEL, and rATP-β Plus rGroEL (rATP-β + rGroEL) Among Two Cats Experimentally Inoculated with Bh and Two Uninoculated Controls Cats

**Figure 1:**
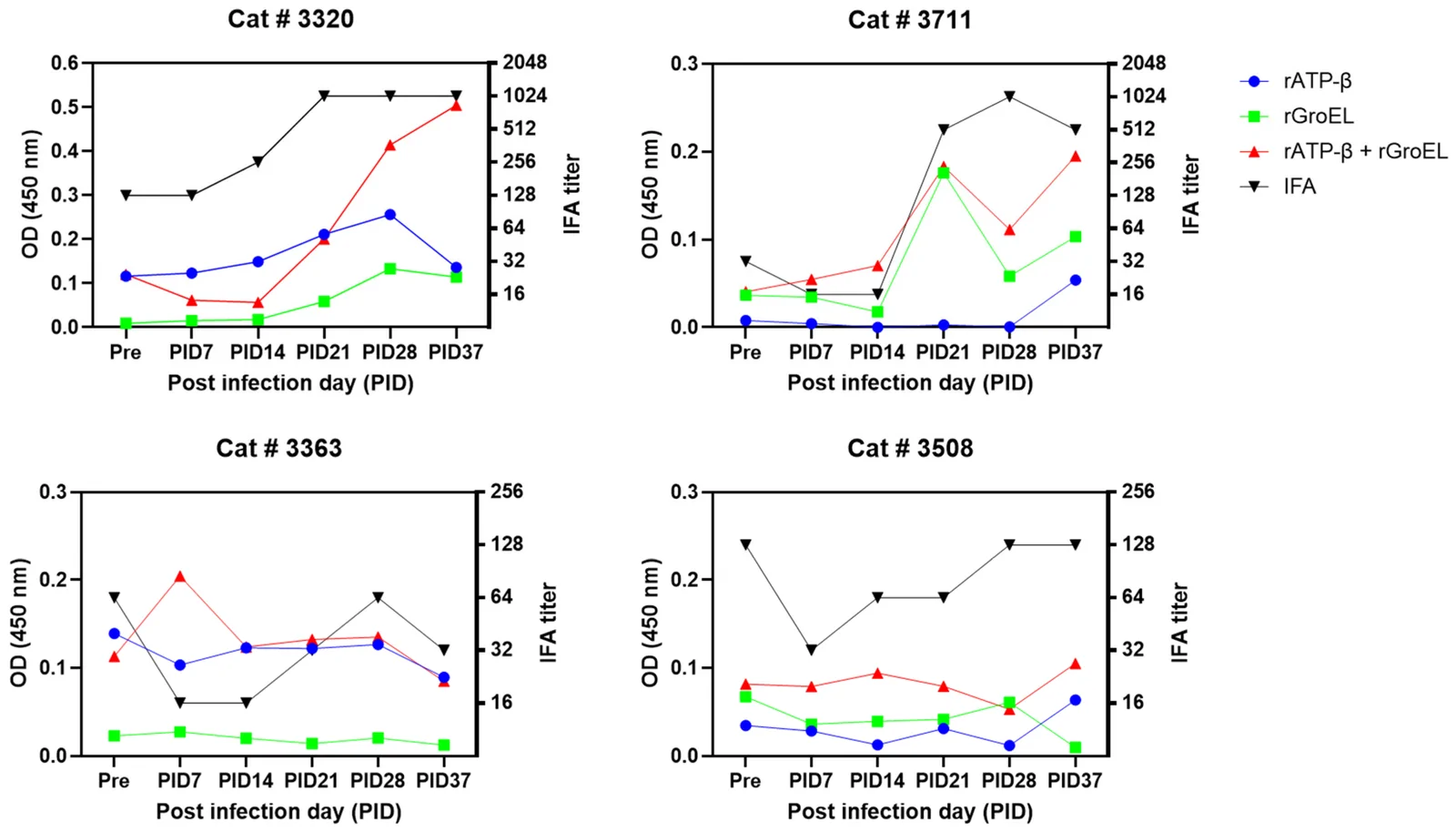
Reactivity of rATP-β, rGroEL, and rATP-β plus rGroEL (combination of rATP-β and rGroEL) among two cats experimentally inoculated with *Bh* (cat# 3320 and # 3711) and two control cats (cat # 3363 and # 3508). Both proteins and the combination were tested by ELISA in triplicate. *Bartonella henselae* IFA titer was plotted on right y-axis of the graph. A cutoff titer of ≥1:64 was used to define an IFA-seropositive titer.

Reactivity of sera from two *Bh*-inoculated cats (cat # 3320 and # 3711) and control cats (cat # 3363 and # 3508) with rATP-β, rGroEL, and rATP-β plus rGroEL was assessed in triplicate by ELISA. Based upon IFA results, prior exposure to a *Bartonella* species was possible in one *Bh*-inoculated cat (cat # 3320) and both control cats as previously described [28]. ELISA reactivity of sera to all proteins (rATP-β, rGroEL, and rATP-β plus rGroEL) increased by more than two-fold following experimental *Bh*-infection (Figure 1 and Supplementary Figure S1). For cat # 3320, reactivity to rGroEL increased 6-fold at 21 days post-inoculation days (PID), 14-fold at PID28, and 12-fold at PID37 when compared with the pre-inoculation time point. Reactivity to rATP-β plus rGroEL increased by more than three-fold at PID28 and PID37 (Figure 1 and Supplementary Figure S1). These findings were consistent with the IFA seroconversion titers for cat # 3320, as the *Bh* IFA titer increased from 1:256 (PID 14) to 1:1024 (PID21, PID28, and PID37). Reactivity of sera from the other *Bh* experimentally inoculated cat (cat # 3711) to rGroEL and rATP-β plus rGroEL increased by more than four-fold by PID 21. For cat # 3711, rATP-β ELISA reactivity was increased seven-fold at PID 37. Among the control cats (cat # 3363 and # 3508), ELISA seroreactivity to proteins rATP-β, rGroEL, and rATP-β plus rGroEL did not exceed a two-fold increase at any post-inoculation time points (Figure 1 and Supplementary Figure S1). Collectively, these results indicate that antibodies to *Bh* rATP-β and rGroEL increased following *Bh*-inoculation in experimentally inoculated cats compared to control cats.

### 3.2. Reactivity Among Group II (Bh qPCR-Positive and IFA-Positive Naturally Infected Cats) and Group III (Bartonella Species qPCR-Negative and IFA-Negative Control Cats)

The sensitivity and specificity of *Bh* rATP-β, rGroEL, and combination of rATP-β and rGroEL (rATP-β plus rGroEL) recombinant proteins were assessed by ELISA using sera from Group II (34 cats naturally infected with *Bh*) and Group III (33 negative control cats). Based on ELISA seroreactivity of the negative control group and receiver operating characteristic (ROC) curve analysis, optimal ELISA optical density (OD) cutoff values were calculated. ELISA OD cutoff values were also selected using the Youden index to optimize sensitivity and specificity for each recombinant protein. Optimal OD cutoff values of 0.1 for the rATP-β and rGroEL ELISAs and 0.17 for the combined rATP-β plus rGroEL ELISA were determined using the Youden index (*J*), taking into account the trade-off between sensitivity and specificity, and were used to evaluate the diagnostic performance of each ELISA. The sensitivity and specificity of each ELISA across a range of OD cutoff values were presented to illustrate the trade-off between them.

**Figure 2:**
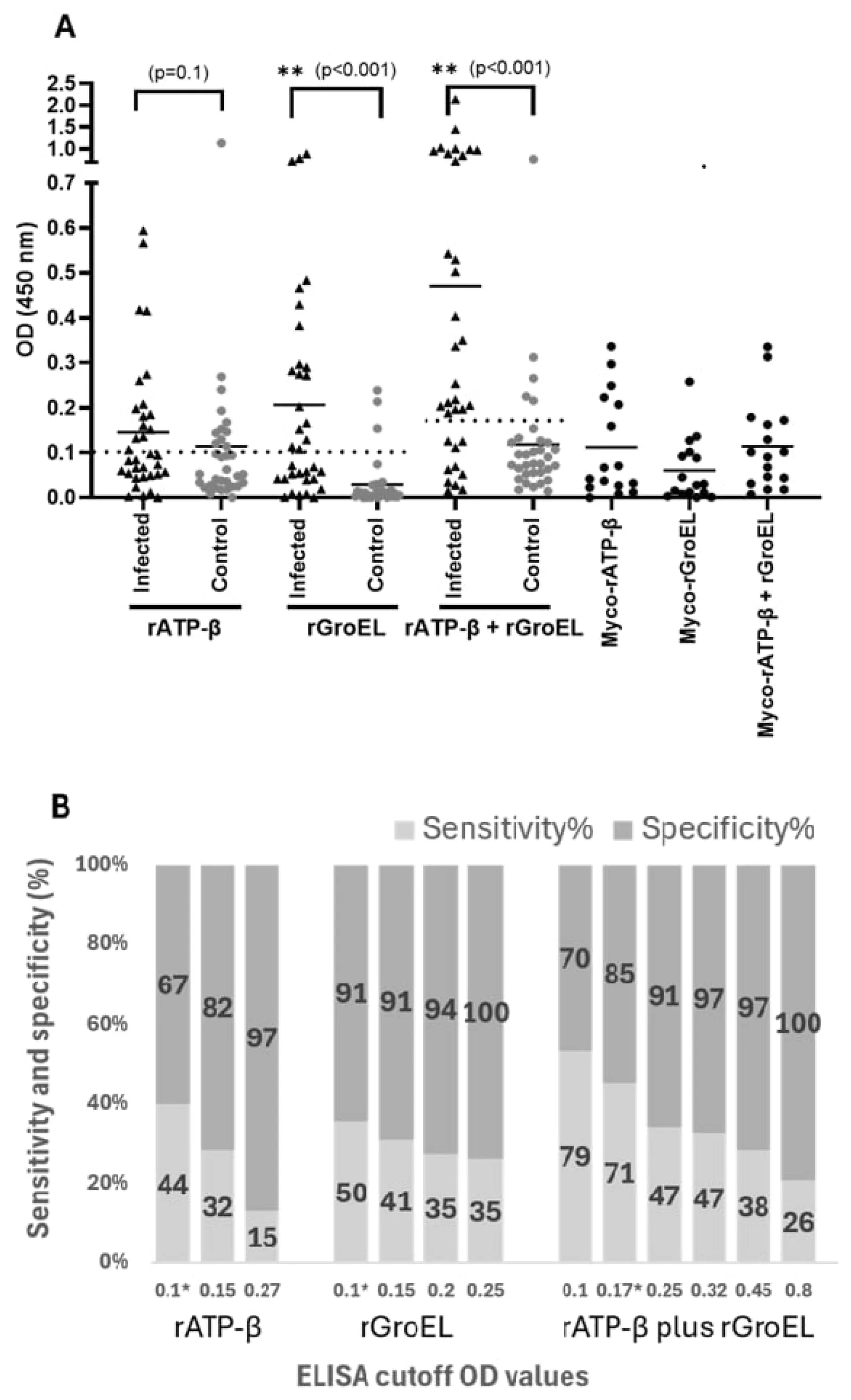
Scatterplots of the ELISA seroreactivity among *Bartonella*-infected and control cats (**A**) and sensitivity and specificity of ELISA for *Bartonella henselae* recombinant rATP-β, rGroEL, and rATP-β plus rGroEL (**B**). For ELISA analysis, sera from cats naturally infected with *Bartonella* spp. (Group II; *n* = 34) and negative control cats (Group III, *n* = 33) were used. The difference in ELISA IgG seroreactivity between the Group II and III cats was determined by the Mann–Whitney U test. Respective *p* values between sample groups are given. Optimal OD cutoff values are represented by the black dotted line. The solid line represents the mean of ELISA OD values for each group. * indicates the optimal ELISA cutoff value for each recombinant protein, determined using Youden index (*J)*; these cutoff values were used to calculate the sensitivity and specificity of each ELISA and its agreement with IFA in this study. Asterisks (**) represent statistical significance (*p *< 0.05, Mann-Whitney U test).

Based on the ELISA reactivity of sera from Group II and Group III cats, the rATP-β-based ELISA resulted in a sensitivity of 44% (95% CI 29–61%) and specificity of 67% (95% CI 50–80%) at an OD cutoff value of 0.1. In contrast, rGroEL ELISA yielded a sensitivity of 50% (95% CI 34–66%) and specificity of 91% (95% CI 76–97%) at an OD cutoff value of 0.1 (Figure 2 and Supplementary Table S1). At a lower OD cutoff value of 0.04 OD, corresponding to the maximal Youden index, the sensitivity and specificity of rATP-β-based ELISA were 85% (95% CI 70–94%) and 42% (95% CI 27–59%), respectively (Figure 2). Similarly, at an OD cutoff value of 0.03, the rGroEL-based ELISA demonstrated a sensitivity of 82% (95% CI: 66–92%) and a specificity of 88% (95% CI: 73–95%) at the maximal Youden index. Lower OD cutoff values of 0.04 and 0.03 for the rATP-β- and rGroEL-based ELISAs, respectively, yielded higher sensitivity but generated more false-positive results, resulting in lower specificity. Because these low OD values are unacceptable for clinical use, a higher OD cutoff value of 0.1 was selected as the final cutoff for the rATP-β- and rGroEL-based ELISAs to improve specificity in this study.

**Figure 3:**
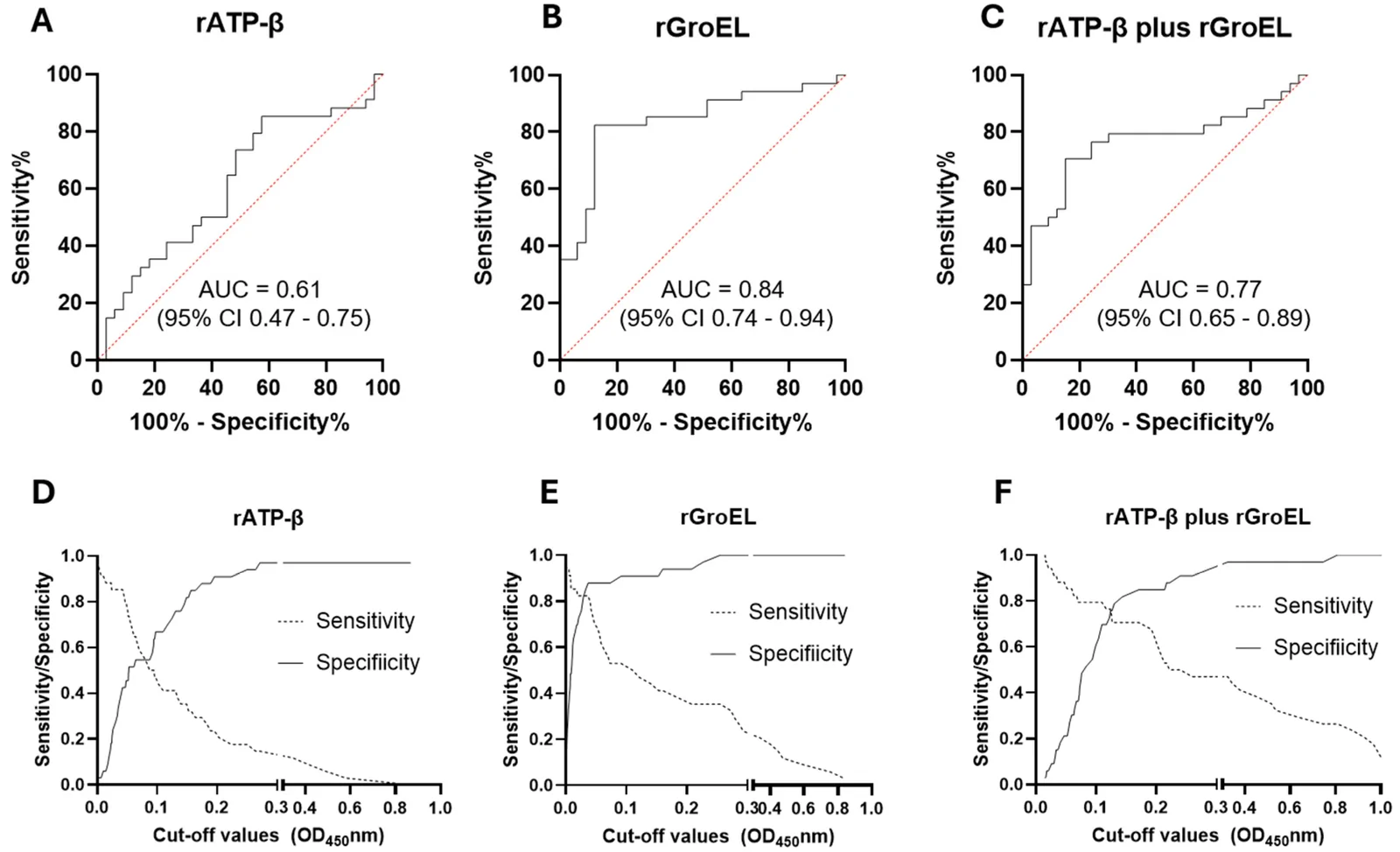
Receiver operating characteristic (ROC) curves with 95% CIs for ELISA seroreactivity of *Bartonella henselae* recombinant proteins for cats. ROC curves for recombinant rATP-β (**A**), rGroEL (**B**), and rATP-β plus rGroEL (**C**). Sensitivity and specificity of rATP-β (**D**), rGroEL (**E**), and rATP-β plus rGroEL (**F**) ELISAs at varying ELISA optical density (OD) cutoff values. For ROC analysis, sera from cats naturally infected with *Bartonella* spp. (Group II; *n* = 34) and negative control cats (Group III, *n* = 33) were used. AUC = area under curve.

Among the 34 Group II cats naturally infected with *Bh*, 19 and 17 cats were tested negative by the rATP-β- and rGroEL- based ELISA, respectively, at the OD cutoff value of 0.1. Of the 33 Group III control cats, eleven and three control cats tested positive by the rATP-β and rGroEL-based ELISA, respectively, at the 0.1 cutoff value. The rATP-β-based ELISA yielded the AUC score 0.61 (95% CI 0.47–0.75) (Figure 3). In comparison, the AUC score for rGroEL-ELISA was 0.84 (95% CI 0.74–0.94), indicating the good discrimination between Group II-positive and Group III-negative sera (Figure 3).

We next assessed the sensitivity and specificity of rATP-β plus rGroEL by combining rATP-β and rGroEL. The sensitivity and specificity of rATP-β plus rGroEL were 71% (95% CI 54–83%) and 85% (95% CI 69–93%) at a cutoff value of 0.17 with the highest Youden Index (*J*) (Figure 2B). Twenty-four (70.6%) Group II cats were seropositive based on the rATP-β plus rGroEL- based ELISA, whereas only five Group III control cats were positive by this combination protein assay (Table 1). The AUC for rATP-β plus rGroEL-based ELISA was 0.77 (95% CI 0.65–0.89), indicating an acceptable ability to distinguish between positive and negative groups (Figure 3). Overall, the reactivity of rGroEL and rATP-β plus rGroEL proteins in Group II infected cats was significantly higher than in Group III control cats (Figure 2, Mann–Whitney U test, *p* < 0.001). However, there was no significant difference in reactivity to rATP-β between sera from infected cats and negative control cats (Figure 2, Mann–Whitney U test, *p* = 0.1). The sensitivity and specificity of the rATP-β-, rGroEL-, and rATP-β plus rGroEL-based ELISAs varied at different ELISA OD cutoff values, as indicated in Figure 3D–F, indicating a trade-off between the sensitivity and specificity of ELISAs for the diagnosis of *Bh* exposure/infection in cats.

### 3.3. Agreement and Correlation Between IFA and ELISA

**Figure 4:**
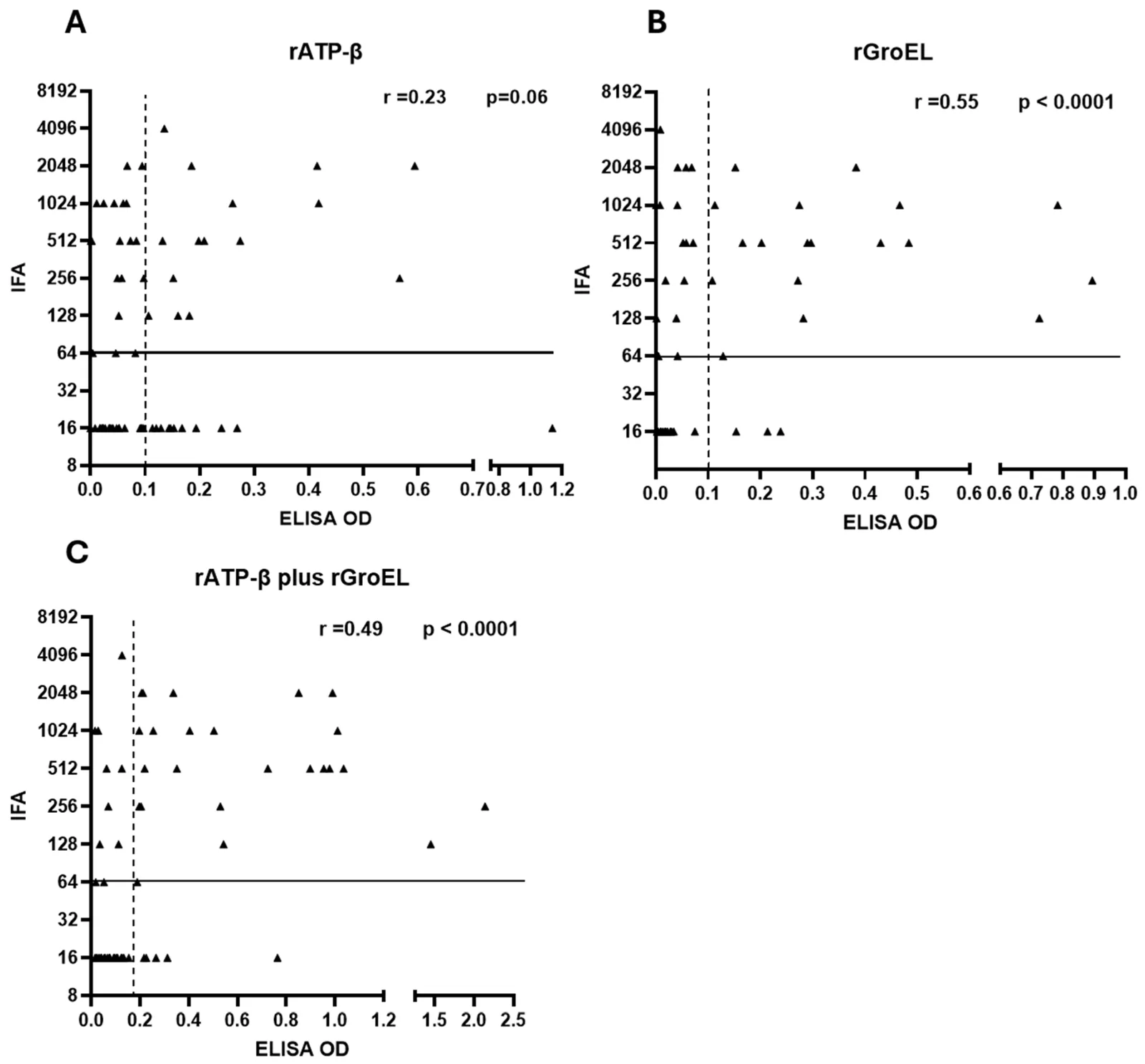
Correlation between *Bartonella henselae* IFA and ELISA optical density results in cats. *Bartonella henselae* (*Bh*) IFA and ELISA results from Group II and Group III cats were used for Spearman correlation (r) analysis. Correlation of *Bh* IFA titers with ELISA OD values for rATP-β (**A**), rGroEL (**B**), and combined rATP-β plus rGroEL (**C**). Group II cats (*n* = 34) were naturally infected with *Bartonella henselae* and Group III cats consisted of 33 *Bartonella* spp. IFA-negative and qPCR-negative control cats. All Group II cats were *Bh* qPCR-positive and IFA-seropositive (IFA titer ≥1:64). The solid vertical lines denote the IFA cutoff titer; the dotted vertical lines represent the ELISA optical density cutoff values.

We next compared the agreement between *Bh* IFA and ELISA results for the rATP-β, rGroEL, and rATP-β plus rGroEL recombinant proteins using Group II naturally infected and Group III negative control cats. Based on Youden index (*J*) and the trade-off between sensitivity and specificity, OD cutoff values of 0.1 for both rATP-β and rGroEL and 0.17 for the combined rATP-β plus rGroEL were selected as optimal for calculating the sensitivity and specificity of each ELISA and its agreement with IFA. The comparisons between IFA and ELISA results are summarized in Table 1. There was moderate agreement between *Bh* IFA and rGroEL-based ELISA (overall agreement 70%; Cohen’s kappa = 0.41) and between *Bh* IFA and rATP-β plus rGroEL-based ELISA (overall agreement 78%, Cohen’s kappa = 0.55) (Table 1). In contrast, agreement between *Bh* IFA and rATP-β was slight (overall agreement 55%, Cohen’s kappa = 0.11). There was a moderate correlation between *Bh* IFA and rGroEL ELISA (Spearman correlation r = 0.55, *p* < 0.0001), and between *Bh* IFA and rATP-β plus rGroEL ELISA results (Spearman correlation r = 0.49, *p* < 0.0001) (Figure 4). *Bartonella henselae* IFA and rATP-β ELISA results were not statistically correlated (Spearman correlation r = 0.23, *p* = 0.06) (Figure 4). Because Group II cats were both IFA- and PCR-positive for *Bartonella henselae*, and Group III cats were both IFA- and PCR-negative for *Bartonella* spp., the agreement between ELISA and PCR status was identical to the agreement between ELISA and IFA described above. Taken together, these findings suggest that agreement between *Bh* IFA/PCR and ELISA was incomplete and varied depending on the evaluated recombinant antigen.

### 3.4. ELISA Seroreactivity of Hemotropic Mycoplasma qPCR-Positive Cats

To further evaluate assay specificity, we tested sera from 16 hemotropic *Mycoplasma* qPCR-positive cats for reactivity with rATP-β, rGroEL, and rATP-β plus rGroEL by ELISA in triplicate. All 16 cats tested negative for *Bartonella* sp. by both qPCR and IFA and negative for NCSU-VBDDL feline comprehensive tests described in the Methods Section. At the optimal cutoff value of 0.1 OD, six cats were seropositive for rATP-β and three were seropositive for rGroEL (Figure 2). Among the three rGroEL-ELISA-positive cats, only one was also seropositive for rATP-β. Using the rATP-β plus rGroEL-based ELISA, two cats were seropositive at the optimal OD cutoff value of 0.17 (Figure 2). Both of these rATP-β plus rGroEL-ELISA-positive cats were also positive by rATP-β-based ELISA but negative by rGroEL-based ELISA (Figure 2).

## 4. Discussion

This study reported the sensitivity and specificity of two *Bh* recombinant proteins, rATP-β and rGroEL, for the serodiagnosis of *Bartonella* infection in cats. To our knowledge, *Bh* rATP-β and rGroEL have not been extensively studied as potential diagnostic candidates for feline bartonellosis. Based on the *Bh* rATP-β, rGroEL, and rATP-β plus rGroEL ELISA results, rGroEL yielded the highest AUC (0.84 (95% CI 0.74–0.94)), followed by rATP-β plus rGroEL with AUC of 0.77 (95% CI 0.65–0.89). The AUC of 0.84 indicates good discrimination between the positive and negative groups by the rGroEL-based ELISA in this study, whereas the AUC of 0.77 indicates acceptable discrimination by the combined rATP-β plus rGroEL ELISA. However, the sensitivity of the rGroEL-based ELISA was only 50% at the optimal OD cutoff value of 0.1, indicating its limited ability to detect anti-*B. henselae* antibodies in infected cats. The rATP-β-based ELISA had a poor AUC (0.61), indicating a limited clinical utility of rATP-β for the serodiagnosis of *Bh* infection/exposure in cats.

At the optimal OD cutoff value of 0.1, the rATP-β- and rGroEL-based ELISAs showed similar sensitivity (44% [95% CI 29–61%] and 50% [95% CI 34–66%], respectively), but the rATP-β-based ELISA had considerably lower specificity (67% [95% CI 50–80%] vs. 91% [95% CI 76–97%]). GroEL belongs to the Hsp60 family of molecular chaperones [36], and the β subunit of ATP synthase (ATP-β) is the catalytic subunit of the enzyme that synthesizes ATP using a proton gradient [37]. Both proteins are highly conserved among eubacteria and may share antigenic epitopes with other eubacteria [36,37]. We therefore selected regions of ATP-β (aa 109–265) and GroEL (aa 193–370) that are conserved across *B. henselae* strains but more variable among other bacteria. GroEL, a heat-shock protein induced in *B. henselae* during stress and infection, is a well-established immunodominant antigen [25,38]. In contrast, ATP-β is a cytoplasmic membrane-associated protein that may be less accessible to the host immune system and may therefore induce a weaker antibody response than GroEL. In addition, the selected rATP-β region may be highly conserved among eubacteria, to which cats are commonly exposed. Consequently, antibodies induced by ATP-β from other bacteria may cross-react with shared or similar epitopes in the selected region of rATP-β. To examine this possibility further, we aligned the selected regions of rATP-β (aa 109–265) and rGroEL (aa 193–370) with *M. haemofelis* sequences using the web-based NCBI protein BLAST portal (https://blast.ncbi.nlm.nih.gov/Blast.cgi). The rATP-β region showed 54% amino acid identity with the corresponding *M. haemofelis* ATP synthase β subunit (GenBank: AEW44994.1), with identical residues distributed throughout the region. By contrast, the rGroEL region showed no significant similarity to *M. haemofelis*, although it shared 58% identity with a GroEL sequence from another *Mycoplasma* species (GenBank: MBD5445954.1). Together, these factors may explain the lower diagnostic performance of the rATP-β-based ELISA.

Because of the low sensitivity of the rATP-β-, rGroEL-, and rATP-β plus rGroEL- based ELISAs, a considerable proportion of *B. henselae*-infected cats may test negative. False-negative results may delay diagnosis, treatment, and disease control, potentially leading to worse clinical outcomes in cats due to complications associated with *Bartonella* infection, such as endocarditis, myocarditis, uveitis, and lymphadenopathy [2,14]. False-negative results may also increase the risk of *B. henselae* transmission among cats and from cats to humans, thereby contributing to the zoonotic spread of *Bartonella* spp. [5]. *Bartonella henselae* is a zoonotic pathogen of One Health importance. *Bartonella* infection can cause clinical manifestations ranging from mild to life-threatening in humans, including bacillary angiomatosis, bacillary peliosis, endocarditis, granulomatous hepatitis, myocarditis, and neuroretinitis [2,6,14]. Accurate and timely diagnosis of infected cats, combined with treatment and flea control, may help prevent the transmission of *Bartonella* spp. among cats and from cats to owners, veterinary personnel, and other cat handlers [1,5]. The diagnostic utility of rATP-β (aa 109–265) and rGroEL (aa 193–370) has not been evaluated for human bartonellosis. Given the limitations of IFA for diagnosing *Bartonella* infection in humans, these antigens may also have potential for human diagnostic applications. However, antibody responses may vary with disease manifestation, immune status, and the infecting *Bartonella* species. In addition, cat, dog, and human antibodies may recognize different epitopes of the same protein. Consequently, sensitivity and specificity may differ between host species. Because of its high specificity in cats, rGroEL could potentially serve as an adjunct tool alongside other serological tests in humans. Future studies are needed to evaluate the diagnostic performance of both antigens for human bartonellosis.

Consistent with the diagnostic performance of *Bh* recombinant proteins for canine bartonellosis in our previous study [26], rGroEL and rATP-β plus rGroEL yielded better overall results than the rATP-β-based ELISA. Collectively, these results suggest that the rGroEL- and rATP-β plus rGroEL-based ELISAs performed well in distinguishing between positive and negative groups. In contrast to the promising diagnostic utility of ATP-β for canine bartonellosis reported in our previous study [26], ATP-β did not demonstrate optimal diagnostic value for feline bartonellosis. Nevertheless, further screening and selection of other antigenic peptides derived from ATP-β and GroEL could improve their respective diagnostic accuracy. Bioinformatic analysis, peptide screening and synthesis, and subsequent evaluation of these and other ATP-β and GroEL peptides for feline bartonellosis may enhance their diagnostic potential, an approach that was beyond the scope of this present study.

A previous study that characterized the antibody response of cats using sera from cats experimentally challenged with *Bh* and from 601 shelter cats, identified at least 24 *Bh* immunodominant proteins, including a 57 kDa protein that was strongly recognized by experimentally infected cats [27]. Among the 601 shelter cats, the 51 and 57 kDa proteins were the most frequently recognized *Bh* immunodominant proteins. Given the reported 30% to >40% *Bh* bacteremic prevalence in shelter and stray cats [27], these 51 and 57 kDa proteins, as recognized by experimentally and naturally infected shelter cats, are most likely *Bh* immunodominant proteins. Based upon comparable molecular weights, it is possible that these 51 and 57 kDa proteins correspond to ATP-β and GroEL, analogous to the proteins identified in our previous study involving dog sera [25,26], which had diagnostic utility for canine bartonellosis. Previous studies investigating the proteome of *Bh* using human sera also identified ATP-β and GroEL as immunodominant proteins [39,40,41]. Furthermore, a study employing a *Bh* genome-wide protein array to profile the antibody response in naturally infected and uninfected cats identified ATP-β (BH15320) and GroEL (BH13530) as differentially reactive antigens, with GroEL being the most reactive [23]. Consistent with the findings in this study, rGroEL appears to be the most sensitive and specific serological target for the diagnosis of feline bartonellosis. A previous study compared the transcriptome of *Bh* 48 h following inoculation of the bacteria into DH82 cells, a canine monocyte-macrophage cell line [38]. The *groEL* RNA transcript was significantly upregulated (2.5-fold change, *p* < 0.05), indicating the potential rapid upregulation of *groEL* during initial infection. The transcriptome data is consistent with the more than two-fold increase in seroreactivity in the two *Bh* experimentally challenged cats (cats # 3320 and # 3711 in this study, Figure 1 and Supplementary Figure S1). An increase in ELISA reactivity to *Bh* rATP-β and rGroEL proteins in sera collected from *Bh* experimentally inoculated cats after inoculation compared with pre-inoculation sera indicated that rATP-β and rGroEL proteins possess immunologically relevant epitopes capable of detecting anti-*Bh* antibodies elicited by the host immune system. These findings further support their potential as diagnostic antigens for feline bartonellosis. However, comprehensive diagnostic evaluation using well-characterized sera from a larger number of experimentally infected cats, confirmed negative animals, and cats with other infections is required to determine assay specificity, cross-reactivity, and overall diagnostic accuracy.

Although IFA is considered the gold-standard assay for the diagnosis of *Bartonella* infection in cats, dogs, and humans [11,16,18,19], the comparison of *Bh* IFA and ELISA in this study documented only a moderate correlation: between *Bh* IFA and rGroEL ELISA (Spearman correlation r = 0.55, *p* < 0.0001), and between *Bh* IFA and rATP-β plus rGroEL ELISA results (Spearman correlation r = 0.49, *p* < 0.0001). Several factors likely contributed to this variability in overall agreement between the two assays, including the selection and preparation of IFA and peptide antigens, assay subjectivity, and differences in the host humoral immune response [18,19,42]. For IFA, *Bh*–inoculated DH82 cells (a canine monocytic macrophage-like cell line) served as the antigen, whereas the ELISA used purified rATP-β, rGroEL, or both. Consequently, the whole-cell IFA detects antibodies directed against a broad range of *Bh* antigens, whereas the ELISA detects only anti-ATP-β and anti-GroEL responses, which could have variable expression due to bacterial strain differences or differences in the host immune response during various stages of infection. Cats that had developed antibodies predominantly against other *Bh* antigens would therefore test positive by IFA but negative by ELISA. Also, cats that failed to mount a strong anti-ATP-β or anti-GroEL response would contribute to the observed disagreement between the assays.

One limitation of this study is the lack of clinical and historical data for the diagnostic serum samples used to assess ELISA sensitivity and specificity. The number of naturally infected cats (Group II, *n* = 34) and negative control cats (Group III = 33) represent a relatively small sample size. Although Group III control cats were *Bartonella* species qPCR-negative and IFA-negative, negative qPCR and IFA results do not rule out prior or current *Bartonella* infection, as previously reported in cat, dog, and human studies [4,7,15,16,43]. This is particularly relevant because all Group II and Group III sera were obtained from sick cats submitted to the NCSU-VBDDL for suspected vector-borne pathogen exposure, including *Bartonella* species. Therefore, some cats classified as controls may have been previously infected with *Bartonella* species despite being IFA/qPCR-negative and hence were misclassified as “negative” controls. Supporting this possibility, previous studies detected *Bartonella* spp. DNA or *Bartonella*-specific antibody titers in specific-pathogen-free (SPF) cats and dogs used in research studies [10,28,43]. In an effort to assess specificity, we evaluated the cross-reactivity and specificity of rATP-β and rGroEL in *Bartonella* sp. qPCR-negative and IFA-negative cats with documented hemotropic *Mycoplasma* infection (qPCR-positive). *Mycoplasma haemofelis* is one of the most prevalent globally distributed hemotropic pathogens commonly found in cats [44,45]. Therefore, sera from cats that tested qPCR-positive for *Mycoplasma haemofelis* were selected for specificity testing. Six, three, and two *Mycoplasma* qPCR-positive cats were reactive to rATP-β, rGroEL, and rATP-β plus rGroEL, respectively. Six of the 16 *Mycoplasma* qPCR-positive cats were positive by the rATP- β ELISA, further highlighting the need for additional epitope mapping and careful selection of the ATP-β region to improve its diagnostic performance for feline bartonellosis. It is also possible that these *Mycoplasma* PCR-positive cats had been previously exposed to *Bartonella* species, as negative *Bartonella* sp. PCR and IFA results do not necessarily rule out prior *Bartonella* exposure, as documented in previous studies [8,46]. Also, cross-reactivity of *Bh* ATP-β and GroEL with antibodies from *Mycoplasma*-positive cats cannot be ruled out. Amino acid sequence similarity between these selected regions of rATP-β (aa 109–265) and rGroEL (aa 193–370) and the corresponding regions in other bacteria, including *Mycoplasma haemofelis*, may contribute to cross-reactivity. Further assessment of potential cross-reactivity using a larger cohort of *Mycoplasma* qPCR-positive cats is needed to draw definitive conclusions regarding the diagnostic specificity of these antigens. Although we assessed the cross-reactivity of rATP-β and rGroEL using sera from *Mycoplasma* qPCR-positive cats, cross-reactivity with sera from cats exposed to other pathogens, for example, *Ehrlichia* spp., *Bordetella* spp., and *Anaplasma* spp., was not evaluated or validated. Further studies are needed to evaluate the potential cross-reactivity of these recombinant proteins with sera from cats exposed to vector-borne pathogens.

## 5. Conclusions

In conclusion, rGroEL and rATP-β plus rGroEL may serve as reliable ELISA antigens for the serodiagnosis of *Bartonella* infection in cats. rGroEL represents a promising antigen for the adjunctive serodiagnosis of feline bartonellosis. Although the rGroEL and rATP-β plus rGroEL-based ELISAs demonstrated promising diagnostic performance, further optimization and validation are necessary to enhance their diagnostic utility in clinical settings so as to achieve greater accuracy and reliability. The sensitivity and specificity of the rGroEL-based ELISA could be further improved through epitope mapping and bioinformatic screening of the antigenic regions of rATP-β and rGroEL. Selected antigenic regions of rATP-β and rGroEL could then be combined with other relevant peptides derived from *Bh* immunodominant proteins. This immunoproteomic strategy may ultimately provide a faster, more practical, and more cost-effective alternative to the labor-intensive reference IFA for the serodiagnosis of feline bartonellosis.

.

## Figures and Tables

**Table 1:** Comparison of *Bartonella henselae* IFA and ELISA results for cats. Group II cats (*n* = 34; naturally infected with *Bartonella henselae*) and Group III cats (*n* = 33; *Bartonella* spp. IFA-negative and PCR-negative control cats) were used for comparison. All Group II cats were *B. henselae* PCR-positive and IFA-seropositive (IFA titer ≥1:64). PPA = positive percentage agreement; NPA= negative percentage agreement; OPA = overall percentage agreement. The optimal OD cutoff values of 0.1 for the rATP-β and rGroEL ELISAs and 0.17 for the combined rATP-β plus rGroEL ELISA were used to calculate the sensitivity and specificity of each ELISA and its agreement with IFA.

**ELISA**	**IFA**		**Sensitivity and Specificity of ELISA**		**Agreement Between IFA and ELISA**		
rATP-B ELISA	**Pos (** ***n*** **= 34)**	**Neg (** ***n*** ** = 33)**	**Sensitivity % (95 CI)**	**Specificity % (95% CI)**	**PPA (95% CI)**	**NPA (95% CI)**	**OPA (95% CI)**
POS	15	11	44 (29–61)	67 (50–80)	44 (29–61)	67 (50–80)	55 (43–67)
NEG	19	22					
rGroEL ELISA							
POS	17	3	50 (34–66)	91 (76–97)	50 (34–66)	91 (76–97)	70 (58–80)
NEG	17	30					
rATP-B plus rGroEL ELISA							
POS	24	5	71 (54–83)	85 (69–93)	71 (54–83)	85 (69–93)	78 (66–86)
NEG	10	28					

## Data Availability

Data supporting the conclusions of this study are included in the article and its Supplementary Materials. The data that support the findings of this study are available from the corresponding author upon request.

## Supplementary Materials

The following supporting information can be downloaded at: https://www.mdpi.com/article/10.3390/ani16213527/s1, Supplementary Figure S1. Reactivity of sera from Group I cats to *Bartonella henselae* rATP-β, rGroEL, and rATP-β plus rGroEL (rATP-β + rGroEL) in fold change relative to sera from pre-inoculation time point. Reactivity of sera from two experimentally inoculated cats with *Bh* (cat # 3320 and # 3711) and control cats (cat # 3363 and # 3508; cat inoculated with saline only) with rATP-β, rGroEL, and rATP-β plus rGroEL were assessed by ELISA in triplicate. Fold change in ELISA seroreactivity was calculated by dividing the average optical density (OD) at each post-inoculation time by the corresponding pre-inoculation time average OD of the same cat (Fold change = post-inoculation OD/pre-inoculation OD). Supplementary Table S1. ELISA reactivity of sera from Group II, Group III, and Group IV cats to *Bartonella henselae* (*Bh*) rATP-β, rGroEL, and rATP-β plus rGroEL (rATP-β + rGroEL). ELISA optical density (OD) readings were measured at 450 nm. For each sample, the average ELISA OD value was calculated from three technical replicates. A cutoff titer of ≥1:64 was used to define an IFA-seropositive titer. Group II: 34 cats naturally infected with *Bh;* Group III: 33 *Bartonella* spp. qPCR-negative and IFA-negative control cats, and Group IV: 16 *Mycoplasma haemofelis* qPCR-positive cats.
