# Supplementary material for: Utility of Bartonella henselae Recombinant Proteins ATP-β and GroEL for Serodiagnosis of Feline Bartonellosis
Source: Animals (Basel). 2026 Nov 7;16(21):3527. doi: 10.3390/ani16213527 (PMC13762069; doi:10.3390/ani16213527)
Supplement: Supplementary file 1 [file animals-16-03527-s001.zip › animals-4588212-supplementary.pdf]

Supplementary Materials

Supplementary Figure S1. Reactivity of sera from Group I cats to *Bartonella henselae* rATP-β, rGroEL, and rATP-β plus rGroEL (rATP-β + rGroEL) in fold change relative to sera from the pre-inoculation time point. Reactivity of sera from two cats experimentally inoculated with *Bh* (cat # 3320 and # 3711) and control cats (cat # 3363 and # 3508; cats inoculated with saline only) with rATP-β, rGroEL, and rATP-β plus rGroEL was assessed by ELISA in triplicate. Fold change in ELISA seroreactivity was calculated by dividing the average optical density (OD) at each post-inoculation time by the corresponding pre-inoculation average OD of the same cat (Fold change = post-inoculation OD/pre-inoculation OD).

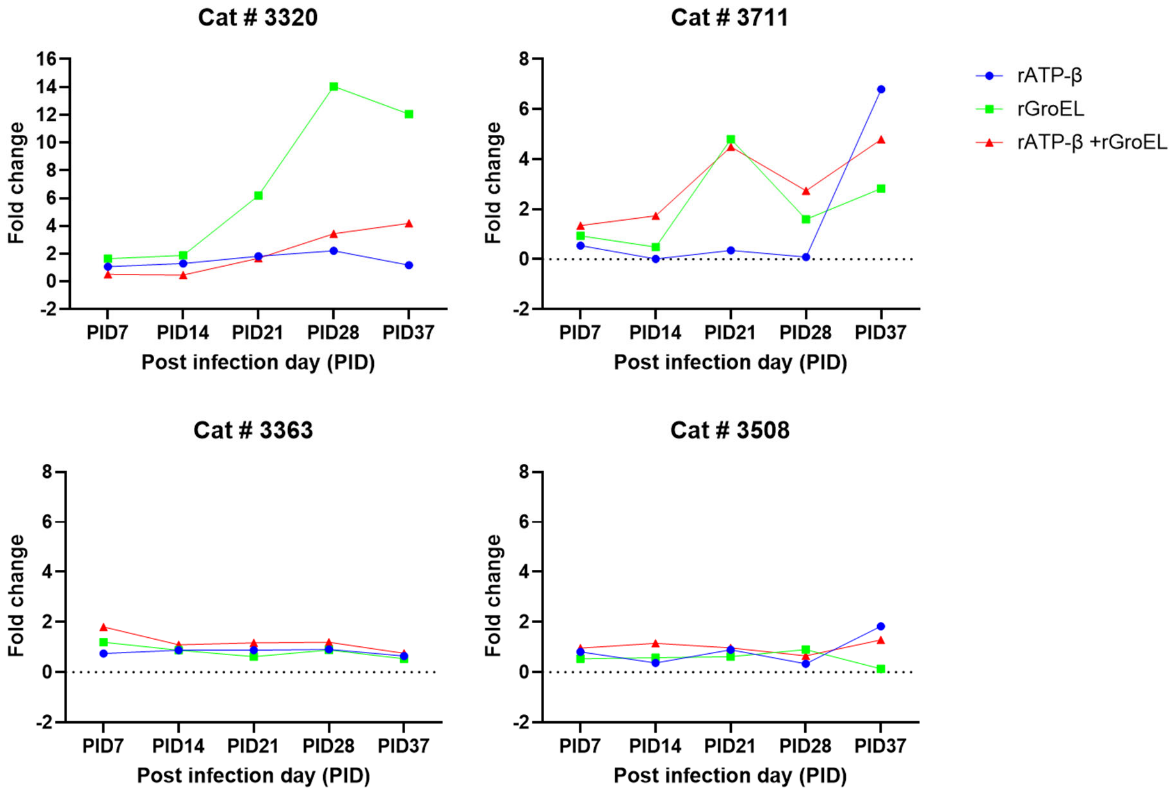

Supplementary Table S1. ELISA reactivity of sera from Group II, Group III, and Group IV cats to *Bartonella henselae* (*Bh*) rATP-β, rGroEL, and rATP-β plus rGroEL (rATP-β + rGroEL). ELISA optical density (OD) readings were measured at 450 nm. For each sample, the average ELISA OD value was calculated from three technical replicates. A cutoff titer of ≥1:64 was used to define an IFA seropositive titer. Group II: 34 cats naturally infected with *Bh*; Group III: 33 *Bartonella* spp. qPCR-negative and IFA-negative control cats; and Group IV: 16 *Mycoplasma haemofelis* qPCR-positive cats.

Cat No.	rATP-β Average OD	rGroEL Average OD	rATP-β + rGroEL Average OD	*Bartonella henselae* IFA Titer
Group II: 34 *Bartonella henselae* qPCR-positive and IFA-positive naturally infected cats				
1	0.060	0.042	0.197	1024
2	0.594	0.152	0.990	2048
3	0.004	0.005	0.018	64
4	0.044	0.113	0.254	1024
5	0.198	0.166	1.035	512
6	0.054	0.058	0.218	512
7	0.002	0.484	0.898	512
8	0.001	0.052	0.125	512
9	0.566	0.894	2.138	256
10	0.274	0.290	0.954	512
11	0.084	0.297	0.724	512
12	0.068	0.042	0.206	2048
13	0.082	0.129	0.188	64
14	0.073	0.071	0.062	512
15	0.260	0.275	0.404	1024
16	0.415	0.058	0.212	2048
17	0.095	0.069	0.336	2048
18	0.098	0.019	0.069	256
19	0.181	0.039	0.112	128
20	0.208	0.202	0.351	512
21	0.012	0.001	0.015	1024
22	0.024	0.008	0.028	1024
23	0.106	0.726	1.450	128
24	0.418	0.466	0.503	1024
25	0.052	0.282	0.543	128
26	0.135	0.009	0.126	4096
27	0.132	0.430	0.978	512
28	0.152	0.055	0.196	256
29	0.057	0.272	0.530	256
30	0.160	0.001	0.035	128
31	0.049	0.108	0.203	256
32	0.066	0.784	1.011	1024
33	0.046	0.042	0.051	64
34	0.185	0.383	0.851	2048

Cat No.	rATP-β Average OD	rGroEL Average OD	rATP-β + rGroEL Average OD	*Bartonella henselae* IFA Titer
Group III: 33 *Bartonella* sp. qPCR-negative and IFA-negative control cats				
1	0.1527	0.0020	0.2653	16
2	0.0382	0.0028	0.0652	16
3	0.0477	0.0017	0.0722	16
4	0.1137	0.0090	0.1537	16
5	0.0272	0.0022	0.0715	16
6	0.0338	0.0048	0.0523	16
7	0.0222	0.0138	0.1073	16
8	0.0003	0.0103	0.0312	16
9	0.0243	0.0003	0.0758	16
10	0.0178	0.0112	0.1328	16
11	0.0360	0.0007	0.0562	16
12	0.0252	0.0075	0.1218	16
13	0.0943	0.0010	0.0732	16
14	0.1438	0.1538	0.0907	16
15	0.1933	0.0280	0.0632	16
16	0.0522	0.0105	0.0320	16
17	0.0213	0.0170	0.0387	16
18	0.0523	0.0053	0.0175	16
19	0.1203	0.0113	0.0555	16
20	0.1290	0.0037	0.0755	16
21	0.2400	0.0280	0.1223	16
22	0.0627	0.0747	0.1268	16
23	1.1382	0.0302	0.7640	16
24	0.0412	0.0065	0.0147	16
25	0.2687	0.0010	0.1022	16
26	0.1675	0.0345	0.2158	16
27	0.0085	0.0195	0.0962	16
28	0.0335	0.0008	0.0237	16
29	0.0910	0.2137	0.3125	16
30	0.0173	0.0037	0.0413	16
31	0.1467	0.0077	0.0963	16
32	0.0932	0.2388	0.2252	16
33	0.0973	0.0227	0.1057	16
Group IV: 16 *Mycoplasma haemofelis* qPCR-positive cats				
1	0.0268	0.0278	0.0435	<1:32
2	0.1588	0.1365	0.1790	<1:32
3	0.3363	0.0117	0.1623	<1:32
4	0.2072	0.0452	0.0678	<1:32
5	0.2228	0.0155	0.1022	<1:32
6	0.2970	0.0890	0.3355	<1:32
7	0.2492	0.0925	0.3133	<1:32
8	0.0668	0.1272	0.0910	1:32

Cat No.	rATP-β Average OD	rGroEL Average OD	rATP-β + rGroEL Average OD	*Bartonella henselae* IFA Titer
9	0.0000	0.0125	0.0072	1:32
10	0.0418	0.1015	0.1015	1:32
11	0.0370	0.0080	0.0308	<1:32
12	0.0710	0.2575	0.1723	<1:32
13	0.0320	0.0013	0.0462	<1:32
14	0.0230	0.0307	0.1290	1:32
15	0.0118	0.0035	0.0180	<1:32
16	0.0085	0.0008	0.0183	<1:32
